# Identification and Validation of an Immune-Related RNA Signature to Predict Survival of Patients With Head and Neck Squamous Cell Carcinoma

**DOI:** 10.3389/fgene.2019.01252

**Published:** 2019-12-04

**Authors:** Shuo Wu, Xinyi Dai, Dielai Xie

**Affiliations:** ^1^Department of E.N.T. & H.N, The Third Affiliated Hospital of Sun Yat-sen University, Guangzhou, China; ^2^School of Life Sciences, Sun Yat-sen University, Guangzhou, China; ^3^Department of Radiology, The Third Affiliated Hospital of Sun Yat-sen University, Guangzhou, China

**Keywords:** head and neck squamous cell carcinoma, biomarkers, prognosis, signature, immune

## Abstract

Head and neck squamous cell carcinoma (HNSCC) is a heterogeneous disease characterized by different molecular subgroups and clinical features. Therefore, it is important to uncover reliable molecular biomarkers for distinguishing different risk patient subgroup. Here, we conducted a multi-omics analysis to examine the joint predictive power of a multi-type RNA signature in the prognosis of HNSCC patients through integration analysis of mRNA, miRNA, and lncRNA expression profiles and clinical data in a large number of HNSCC patients. A multi-type RNA signature (15SigRS) was constructed which can classify patients into the high-risk group and low-risk group with the significantly different outcome [hazard ratio (HR) = 2.718, 95% confidence interval (CI), 2.258–3.272, p < 0.001] in the discovery data set, and subsequently validated in the Cancer Genome Atlas (TCGA) testing data set (HR = 1.299, 95% CI, 1.170–1.442, p < 0.001) and another independent GSE65858 data set (HR = 1.077, 95% CI, 1.016–1.143, p = 0.013). Further multivariate Cox regression analysis and stratification analysis demonstrated the independence of predictive performance of the 15SigRS relative to conventional clinicopathological factors. Furthermore, the 15SigRS has a prior performance in prognostic prediction than other single RNA type-based signatures. Functional analysis suggested that the 15SigRS are involved in immune- or metabolism-related KEGG pathways. In summary, our study demonstrated the potential application of mixed RNA types as molecular markers for predicting the outcome of cancer patients.

## Introduction

Head and neck squamous cell carcinoma (HNSCC), the most frequent histological type of head and neck cancers, is the sixth most common cancers worldwide and account for nearly 5% of all malignancies worldwide (Marur and Forastiere, 2016). Smoking tobacco, drinking alcohol, and human papillomaviruses (HPV) are important risk factors and have been implicated in the pathogenesis of HNSCC (Kobayashi et al., 2018). Surgery combined with radiation therapy, chemotherapy, and targeted therapy is the main treatment option. Although TNM stage has been considered as an important clinical prognostic factor for guiding treatment options, some patients with the same clinical features may have different prognosis because of molecular heterogeneity. Therefore, there is an urgent need to identify reliable biomarkers for predicting prognosis of HNSCC patients

With advances in high-throughput omics technique, increasing efforts have been made to meet this urgent need. Some previous studies used gene expression data and identified some mRNA-based signatures. For example, Bai and colleagues identified a 12-gene signature for predicting progression and prognosis (Bai et al., 2019) Another six-mRNA signature was identified by Tian et al. to predict the death risk of HNSCC patients using gene expression profiles in the Cancer Genome Atlas (TCGA) (Tian et al., 2019). Recently, non-coding RNAs (ncRNAs) have been found to be an important class of RNA molecules and are involved a wide range of biological processes (Fatica and Bozzoni, 2014; Bracken et al., 2016). The dysregulation of ncRNAs has been implicated in various human diseases including cancers (Esteller, 2011), demonstrating the role of ncRNAs as a potential biomarker in cancer diagnosis, prognosis, and treatment (Li et al., 2014; Gonzalez et al., 2015; Jiang et al., 2016; Zhou et al., 2017; Zhou et al., 2018a; Zhou et al., 2018b; Zhou et al., 2019). For HNSCC, recent studies have revealed the altered expression of ncRNAs in the development and progression of HNSCC (Salyakina and Tsinoremas, 2016; Sannigrahi et al., 2018), and several miRNA- or lncRNA-related signatures were identified to improve clinical outcome (Irani, 2016; Wong et al., 2016; Cao et al., 2017; Liu et al., 2018; Diao et al., 2019). However, previous signatures often focus on one type of RNAs, and the joint predictive power of multiple types of RNAs was not investigated yet.

In this study, we tried to investigate the joint predictive power of multi-type RNAs as novel prognostic biomarkers by integrating mRNA expression profiles, miRNA expression profiles, lncRNA expression profiles, and clinical data in a large number of HNSCC patients.

## Materials and Methods

### Patient Data Set

RNA-Seq data (HTSeq), miRNA expression data (Illumina HiSeq), and corresponding clinical data were derived from the TCGA database (https://cancergenome.nih.gov/). Ensembl gene id of mRNAs, miRNAs, and lncRNAs were derived from HUGO Gene Nomenclature Committee (HGNC) database (https://www.genenames.org/). After cross-referenced by Ensembl gene id and tumor barcodes and removing patient samples without survival information and genes with zero expression values in more than 10% samples, a total of 19,163 mRNAs, 3,931 lncRNAs, and 1,854 miRNAs in 489 patients were obtained. All patients were randomly split into two equal patient cohorts: discovery data set (n = 245) and validation data set (n = 244). Another independent validation data set including 270 HNSCC patients was obtained from the Gene Expression Omnibus (GEO) database under the accession number GSE65858 (https://www.ncbi.nlm.nih.gov/geo/query/acc.cgi?acc=GSE65858). Clinical features of HNSCC patients used in this study can be seen in **Table 1**.

**Table 1:** Summary of clinical characteristics of three HNSCC patient data sets in the study.

Characteristic		Discovery dataset (N = 245)	Validation dataset (N = 244)	TCGA dataset (N = 489)	GSE65858 dataset (N = 270)
Vital status, n (%)	Alive	150 (61.2)	128 (52.5)	278 (56.9)	176 (65.2)
	Dead	95 (38.8)	116 (47.5)	211 (43.1)	94 (34.8)
Age (years), n (%)	> = 60	132 (53.9)	141 (57.8)	273 (55.8)	117 (49.3)
	<60	113 (46.1)	103 (42.2)	216 (44.2)	153 (56.7)
Gender, n (%)	Female	64 (26.1)	66 (27.0)	130 (26.6)	47 (17.4)
	Male	181 (73.9)	178 (73.0)	359 (73.4)	223 (82.6)
Stage, n (%)	Stage I/II	57 (23.3)	37 (15.2)	94 (19.2)	55 (20.4)
	Stage III/IV	157 (64.1)	171 (70.1)	328 (67.1)	215 (79.6)
	NA	31 (12.6)	36 (14.7)	67 (13.7)	
Grade, n (%)	G1	29 (11.8)	32 (13.1)	61 (12.5)	
	G2	147 (60)	144 (59.0)	291 (59.5)	
	G3	59 (24.1)	58 (23.8)	117 (23.9)	
	NA	10 (4.1)	10 (4.1)	20 (4.1)	
Race, n (%)	White	201 (82)	216 (88.5)	417 (85.3)	
	Other_race	34 (13.9)	24 (9.8)	58 (11.9)
	NA	10 (4.1)	4 (1.7)	14 (2.9)	
ANGIOLYMPHATIC_INVASION, n (%)	Yes	54 (22)	61 (25)	115 (23.5)	
	No	112 (45.7)	104 (42.6)	216 (44.2)	
	NA	79 (32.2)	79 (32.4)	158 (32.3)	
PERINEURAL_INVASION, n (%)	Yes	72 (29.4)	86 (35.2)	158 (32.3)	
	No	98 (40)	86 (35.2)	184 (37.6)	
	NA	75 (30.6)	72 (29.5)	147 (30.1)	
Smoking_pack_years, n (%)	> = 40	82 (33.5)	78 (32.0)	160 (32.7)	222 (YES, 82.2)
	<40	61 (24.9)	58 (23.8)	119 (24.3)	48 (NO, 17.8)
	NA	102 (41.6)	108 (44.2)	210 (42.9)	
ALCOHOL_HISTORY_DOCUMENTED, n (%)	Yes	159 (64.9)	165 (67.6)	324 (66.3)	
	No	81 (33.1)	73 (29.9)	154 (31.5)	
	NA	5 (2)	6 (2.5)	11 (2.2)	
HPV_STATUS_P16, n (%)	Negative	37 (15.1)	32 (13.1)	69 (14.1)	
	Positive	15 (6.1)	15 (6.1)	30 (6.1)	
	NA	193 (78.8)	197 (80.8)	390 (79.8)	

HNSCC, head and neck squamous cell carcinoma; TCGA, the Cancer Genome Atlas.

### Identification of Survival-Related a Multi-Type RNA Prognostic Signature

To identify survival-related genes, univariate Cox proportional hazards analyses were used to identify candidate prognostic mRNAs, miRNAs, and lncRNAs. Candidate prognostic mRNAs, miRNAs, and lncRNAs were retained only if they have significant *p* values (*p* < 0.05). Then these candidate prognostic mRNAs, miRNAs, and lncRNAs were fitted in a multivariable Cox regression analysis to identify independent survival-related genes. Finally, multi-type RNA prognostic signature was constructed as the linear combination of expression values of each independent survival-related mRNAs, miRNAs, and lncRNAs, weighted by their estimated regression coefficients in the multivariate Cox regression analysis according to previous studies (Zhou et al., 2015a; Zhou et al., 2015b).

### Statistical Analysis

Kaplan–Meier survival curve analysis and a log-rank test were used to compare differences in overall survival (OS) time between the high-risk group and low-risk group. Univariate and multivariate Cox regression analyses were performed on the individual clinical variables with and without the multi-type RNA prognostic signature in each data set. Hazard ratios (HRs) and 95% confidence intervals (CIs) were calculated. The time-dependent receiver operating characteristic (ROC) curve at 3 and 5 years was then calculated to compare the sensitivity and specificity of survival prediction. Hierarchical clustering of the expression values of independent prognostic gene biomarkers was performed using the metric of Euclidean distance and complete linkage. The chi-square test was used to test the significance of survival status between two groups. All statistical analyses were performed using the R/Bioconductor (version 3.0.2).

### Functional Enrichment Analysis

GO and KEGG functional enrichment analysis was performed using Bioconductor package “clusterProfiler” (Yu et al., 2012).

## Results

### Identification of Independent Survival-Related mRNAs, miRNAs, and lncRNAs

**Figure 1:**
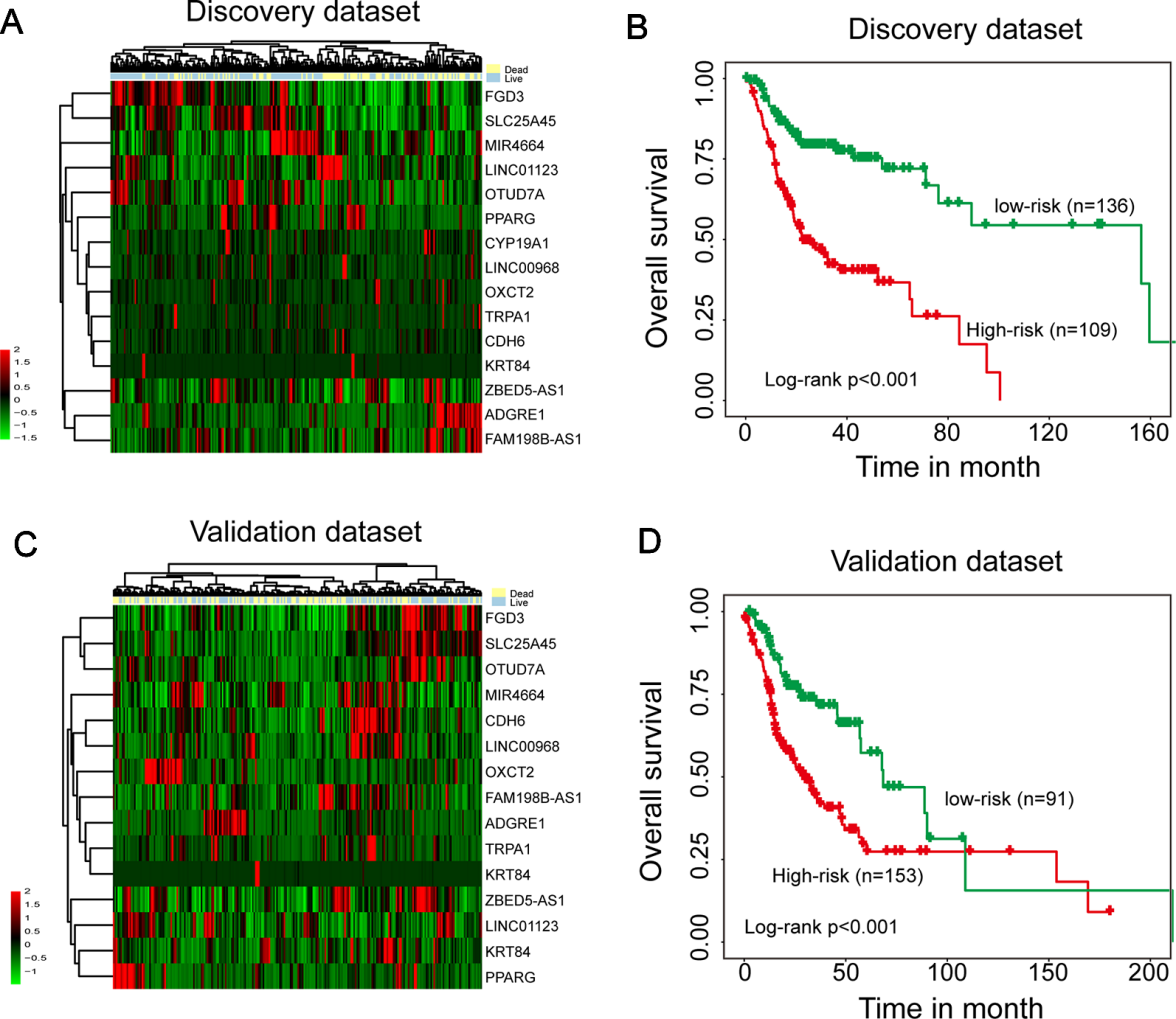
Identification of independent survival-related mRNAs, miRNAs, and lncRNAs. **(A)** Hierarchical clustering analysis of 245 patients in the discovery data set using 15 prognostic genes. **(B)** Kaplan–Meier survival curves of overall survival between two clusters in the discovery data set. **(C)** Hierarchical clustering analysis of 244 patients in the validation data set using 15 prognostic genes. **(D)** Kaplan–Meier survival curves of overall survival between two clusters in the validation data set.

To identify survival-related mRNAs, miRNAs, and lncRNAs, we performed univariate Cox regression analysis to evaluate the association between expression of each type of RNA and OS in the discovery data set. A total of 23 mRNAs, 15 lncRNAs, and 1 miRNAs were found to be significantly associated with OS, and were considered as candidate prognostic mRNAs, miRNAs, and lncRNAs. Then all these candidate prognostic mRNAs, miRNAs, and lncRNAs were fitted into multivariate Cox regression analysis, 15 of 39 genes were identified as independent prognostic gene biomarkers. Hierarchical clustering of the expression values of 15 independent prognostic gene biomarkers revealed two distinctive sample clusters in the discovery data set (**Figure 1A**). The survival status of two distinctive sample clusters is significantly different (dead 57.8% vs. 23.5%, p = 9.349e^-08^, chi-square test). Survival analysis suggested that the OS time between the two sample clusters was significantly different (**Figure 1B**, p < 0.001, log-rank test). Similar results also were observed in the validation data set. Two distinctive sample clusters also were obtained using hierarchical clustering analysis (**Figure 1C**). These two distinctive sample clusters have significantly different survival status (dead 55.5% vs. 34.1%, p = 0.002, chi-square test) and survival time (**Figure 1D**, p < 0.001, log-rank test). These results revealed the potential of these 15 candidate independent prognostic genes as biomarkers in the prognosis of HNSCC patients.

### Establishment and Evaluation of a Multi-Type RNA Prognostic Signature in Predicting Survival in the Discovery Data Set

**Figure 2:**
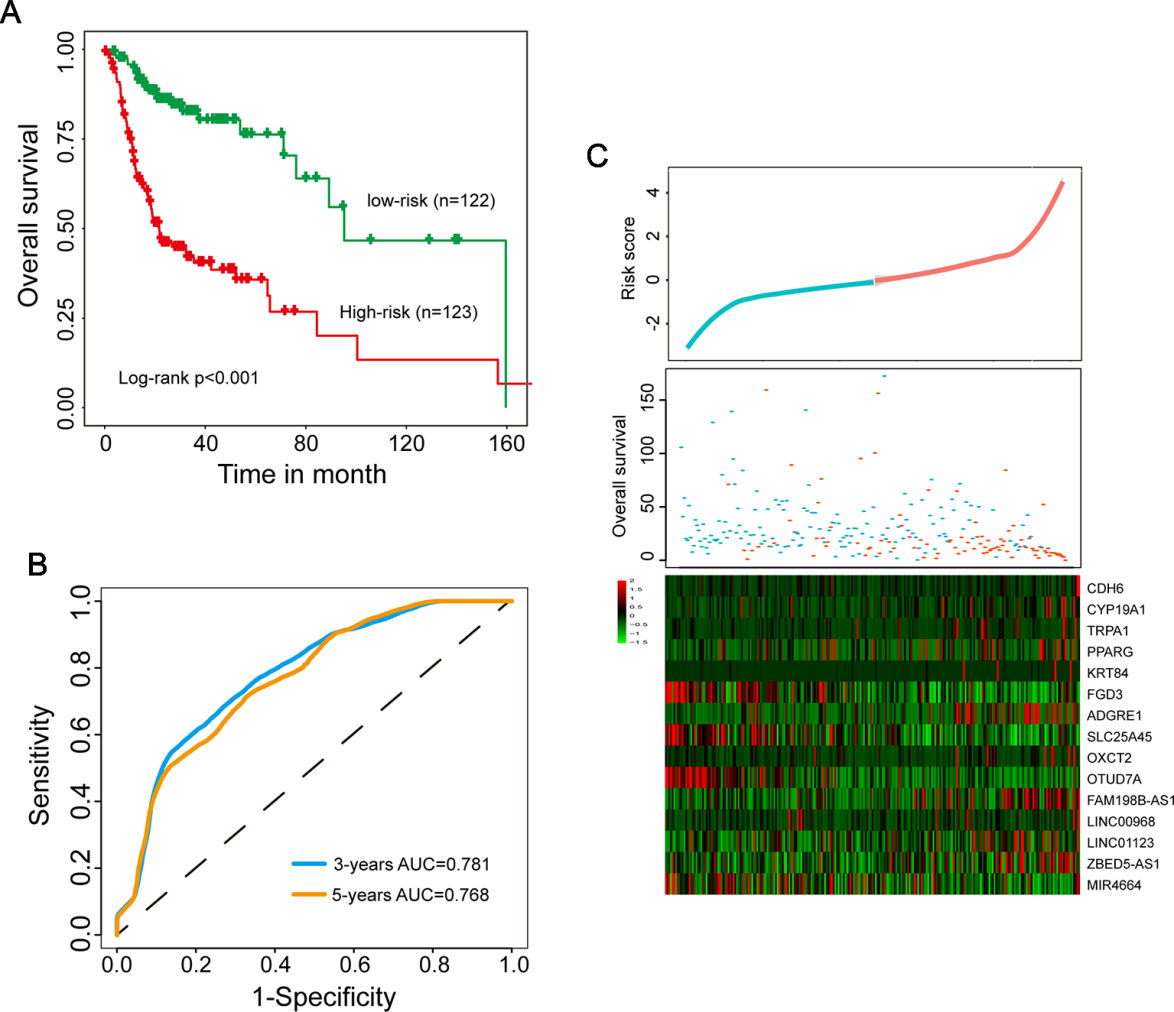
Development and evaluation of the 15SigRS in the discovery data set. **(A)** Kaplan–Meier survival curves of overall survival between the high-risk group and low-risk group. **(B)** Time-dependent receiver operating characteristic (ROC) analysis at 3 and 5 years. **(C)** The distribution of risk scores and survival status of patients and expression patterns of 15 prognostic genes in the 15SigRS.

To establish a multi-type RNA prognostic signature for survival prediction, these 15 candidate independent prognostic genes were fitted in a multivariate Cox regression analysis in the discovery data set. Then a multi-type RNA prognostic signature (15SigRS) were constructed according to the expression of 15 prognostic genes and multivariate Cox regression coefficient as the weight using risk scoring method as described previously, as follows: 15SigRS = (0.5344*CDH6)+(1.0462*CYP19A1)+(0.4723*TRPA1)+(0.2764*PPARG)+(0.0068*KRT84)+(−0.2291*FGD3)+(0.3113*ADGRE1)+(−0.7948*SLC25A45)+(0.4878*OXCT2)+(−4.0659*OTUD7A)+(1.2231*FAM198B-AS1)+(−0.3978*LINC00968)+(1.8352*LINC01123)+(0.1240*ZBED5-AS1)+(−0.0602*MIR4664). We computed a 15SigRS for each HNSCC patient and classified patients into the high-risk group or low-risk group with the cutoff point of median risk score (−0.04) in the discovery data set. Using the 15SigRS, 245 patients in the discovery data set were divided into high-risk (n = 123) and low-risk groups (n = 122). We found that the survival time of the high-risk group is significantly shorter than the low-risk group (**Figure 2A**, p < 0.001, log-rank test). The time-dependent ROC curves analysis for the15SigRS achieved an area under the ROC curve (AUC) of 0.781 at 3 years and 0.768 at 5 years (**Figure 2B**). The distribution of risk scores and survival status of patients and expression patterns of 15 prognostic genes in the 15SigRS were shown in **Figure 2C**.

### Independent Confirmation of the 15SigRs for Survival Prediction in the Validation Data Set and TCGA Data Set

**Figure 3:**
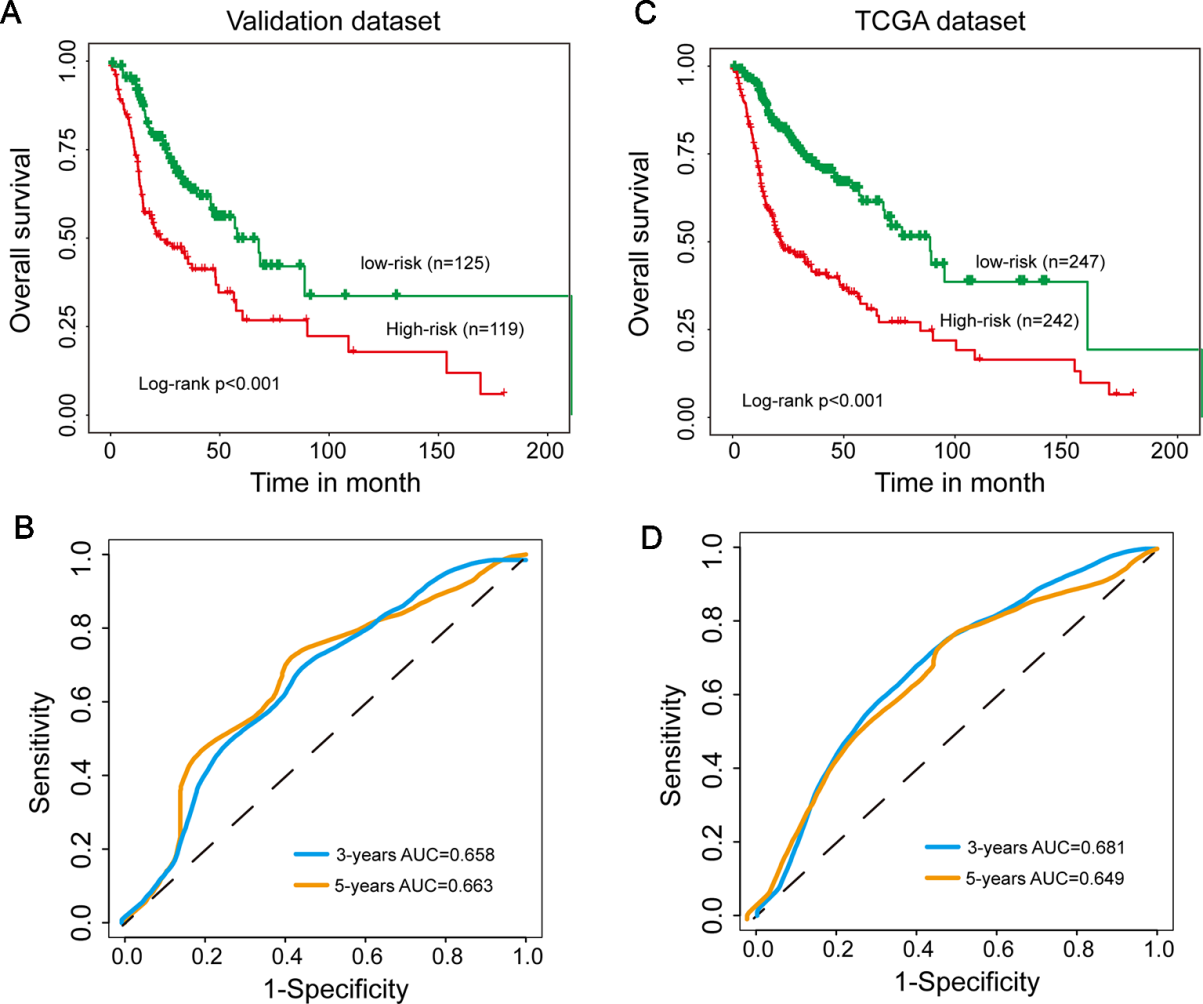
Independent validation of the 15SigRS in the Cancer Genome Atlas (TCGA) data set. **(A)** Kaplan–Meier survival curves of overall survival between the high-risk group and low-risk group in the validation data set. **(B)** Time-dependent ROC analysis at 3 and 5 years in the validation data set. **(C)** Kaplan–Meier survival curves of overall survival between the high-risk group and low-risk group in the TCGA data set. **(D)** Time-dependent ROC analysis at 3 and 5 years in the TCGA data set.

To evaluate the robustness of prognostic performance of the15SigRS, the 15SigRS was tested in the independent validation data set. With 15SigRS and cutoff derived from the discovery data set, all 244 patients in the validation data set also were classified into the high-risk group (n = 119) and low-risk group (n = 125). As shown in **Figure 3A**, patients in the low-risk group showed a better outcome than those in the high-risk group (**Figure 3A**, p < 0.001, log-rank test). The time-dependent ROC curves analysis for the15SigRS achieved an AUC of 0.658 at 3 years and 0.663 at 5 years (**Figure 3B**). In univariate analysis, the HRs of high-risk group versus low-risk group for OS were 1.299 (p < 0.001, CI, 1.170–1.442) (**Table 2**).

**Table 2:** Univariate and multivariate Cox regression analysis of OS in each data set.

Variable	Univariate analysis			Multivariable analysis		
	HR	95% CI of HR	P value	HR	95% CI of HR	P value
**Discovery data set (n = 245)**						
15SigRS	2.718	2.258–3.272	<0.001	2.562	1.999–3.284	<0.001
Age	1.043	1.024–1.063	0.000	1.016	0.991–1.042	0.218
Gender (male/female)	0.643	0.42–0.984	0.042	0.499	0.272–0.916	0.025
Stage (III&IV/I&II)	1.254	0.753–2.088	0.385	1.187	0.352–4.001	0.783
Grade (G2/G1)	1.362	0.703–2.640	0.360	1.685	0.584–4.861	0.334
Grade (G3/G1)	1.139	0.554–2.343	0.723	1.829	0.599–5.582	0.289
Race (White/other race)	0.578	0.329–1.014	0.056	1.623	0.679–3.880	0.276
ALCOHOL_HISTORY_DOCUMENTED (yes/no)	0.734	0.483–1.113	0.145	1.010	0.562–1.815	0.974
ANGIOLYMPHATIC_INVASION (yes/no)	1.687	0.988–2.881	0.056			
PERINEURAL_INVASION (yes/no)	2.879	1.689–4.907	0.000			
SMOKING_PACK_YEARS	1.001	0.995–1.008	0.737			
**Validation data set (n = 244)**						
15SigRS	1.299	1.170–1.442	<0.001	1.311	1.158–1.484	<0.001
Age	1.003	0.986–1.02	0.721	1.020	0.996–1.045	0.111
Gender (male/female)	0.904	0.605–1.352	0.624	1.063	0.606–1.866	0.832
Stage (III & IV/I & II)	3.294	1.595–6.805	0.001	3.102	0.569–16.905	0.191
Grade (G2/G1)	2.264	1.157–4.430	0.017	1.326	0.593–2.967	0.492
Grade (G3/G1)	1.927	0.936–3.964	0.075	1.248	0.520–2.993	0.620
Race (White/other race)	0.871	0.477–1.587	0.651	0.900	0.452–1.790	0.763
ALCOHOL_HISTORY_DOCUMENTED (yes/no)	1.185	0.788–1.78	0.415	1.536	0.882–2.675	0.130
ANGIOLYMPHATIC_INVASION (yes/no)	1.809	1.138–2.874	0.012			
PERINEURAL_INVASION (yes/no)	1.698	1.061–2.716	0.027			
SMOKING_PACK_YEARS	1.001	0.992–1.011	0.801			
**TCGA data set (n = 489)**						
15SigRS	1.496	1.393–1.606	<0.001	1.482	1.348–1.629	<0.001
Age	1.022	1.009–1.035	0.001	1.027	1.010–1.044	0.002
Gender (male/female)	0.759	0.568–1.016	0.064	0.786	0.526–1.174	0.239
Stage (III & IV/I & II)	1.812	1.216–2.701	0.003	1.846	0.768–4.437	0.171
Grade (G2/G1)	1.749	1.102–2.777	0.018	1.240	0.679–2.264	0.483
Grade (G3/G1)	1.507	0.913–2.487	0.109	1.441	0.754–2.754	0.269
Race (White/other race)	0.710	0.473–1.065	0.098	0.811	0.492–1.335	0.410
ALCOHOL_HISTORY_DOCUMENTED (yes/no)	0.951	0.712–1.27	0.734	1.165	0.792–1.714	0.437
ANGIOLYMPHATIC_INVASION (yes/no)	1.750	1.239–2.473	0.001			
PERINEURAL_INVASION (yes/no)	2.222	1.563–3.16	0.000			
SMOKING_PACK_YEARS	1.001	0.995–1.006	0.765			
HPV_STATUS_P16 (yes/no)	0.504	0.172–1.477	0.212			
**GSE65858 data set (n = 270)**						
15SigRS	1.077	1.016–1.143	0.013	1.073	1.012–1.137	0.019
Age	1.037	1.006–1.048	0.012	1.03	1.007–1.053	0.01
Gender (male/female)	1.046	0.6174–1.771	0.868	1.026	0.602–1.749	0.923
Stage (II/I)	0.386	0.112–1.333	0.132	0.306	0.088–1.071	0.064
Stage (III/I & II)	0.459	0.1447–1.454	0.185	0.423	0.133–1.343	0.144
Stage (IV/I&)	1.495	0.603–3.705	0.385	1.339	0.537–3.336	0.531
SMOKING (yes/no)	0.941	0.555–1.595	0.821	1.294	0.733–2.284	0.373

OS, overall survival; HR, hazard ratio.

A similar analysis also was performed in the TCGA data set. The patients of TCGA data set were segregated into a high-risk group (n = 242) and low-risk group (n = 247) with significantly different OS (**Figure 3C**, p < 0.001, log-rank test). The time-dependent ROC curves analysis for the15SigRS achieved an AUC of 0.681 at 3 years and 0.649 at 5 years (**Figure 3D**). In univariate analysis, the HRs of high-risk group versus low-risk group for OS were 1.496 (p < 0.001, CI, 1.393–1.606) (**Table 2**).

### Further Confirmation of the 15SigRs for Survival Prediction in GEO Data Set With Microarray Platform

**Figure 4:**
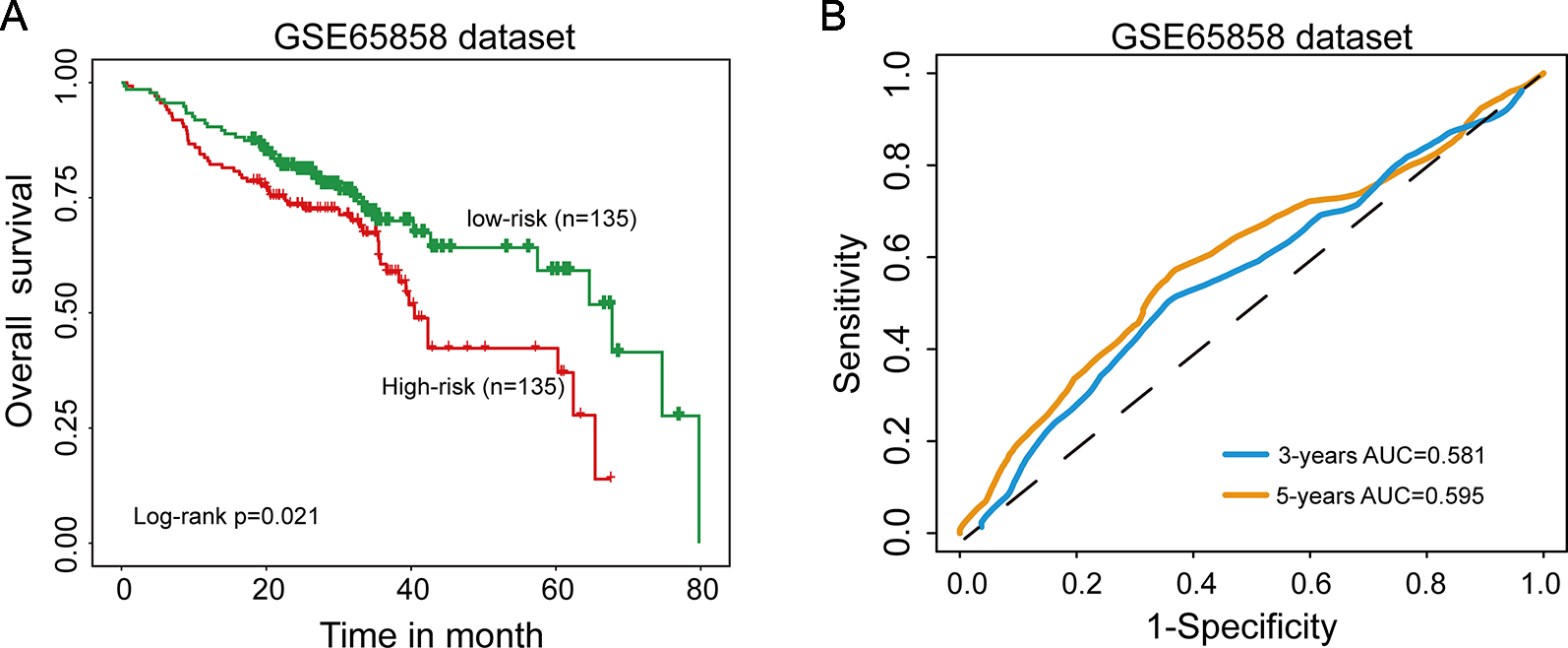
Independent validation of the 15SigRS in the Gene Expression Omnibus (GEO) data set. **(A)** Kaplan–Meier survival curves of overall survival between the high-risk group and low-risk group in the GSE65858 data set. **(B)** Time-dependent ROC analysis at 3 and 5 years in the GSE65858 data set.

Further validation of the 15SigRS for survival prediction was performed using another independent data set (GSE65858) of 270 patients with microarray platform (Illumina HumanHT-12 V4.0). Finally, expression value of 9 mRNAs of the 15SigRS can be obtained from GSE65858. With the same score model, the 15SigRS could distinguish between patients with high and low risks of death (**Figure 4A**, p = 0.021, log-rank test). The OS rate of patients in the low group were 69.9% at 3 years and 59.2% at 5 years, respectively, which is significantly higher than that (60.6% at 3 years and 37% at 5 years) in the high-risk group. The AUC of time-dependent ROC curves analysis is 0.581 at 3 years and 0.595 at 5 years (**Figure 4B**). In univariate analysis, the HRs of high-risk group versus low-risk group for OS were 1.077 (p = 0.013, CI, 1.016–1.143) (**Table 2**).

### Independent Predictive Power of the 15SigRs From Clinicopathological Factors

To further investigate whether the predictive power of the 15SigRS was independent of other clinicopathological factors, we performed multivariate Cox regression analysis of the15SigRS with selected covariables including age, gender, stage, grade, race, and alcohol history. Results of multivariate analysis suggested that the 15SigRS still have a significant association with OS when adjusted by other clinicopathological factors in the discovery data set (HR = 2.562, p < 0.001; 95% CI, 1.999–3.284), validation data set (HR = 1.311, p < 0.001; 95% CI, 1.158–1.484), TCGA data set (HR = 1.482, p < 0.001; 95% CI, 1.348–1.629), and independent GSE65858 data set (HR = 1.073, p = 0.019; 95% CI, 1.012–1.137) (**Table 2**).

**Figure 5:**
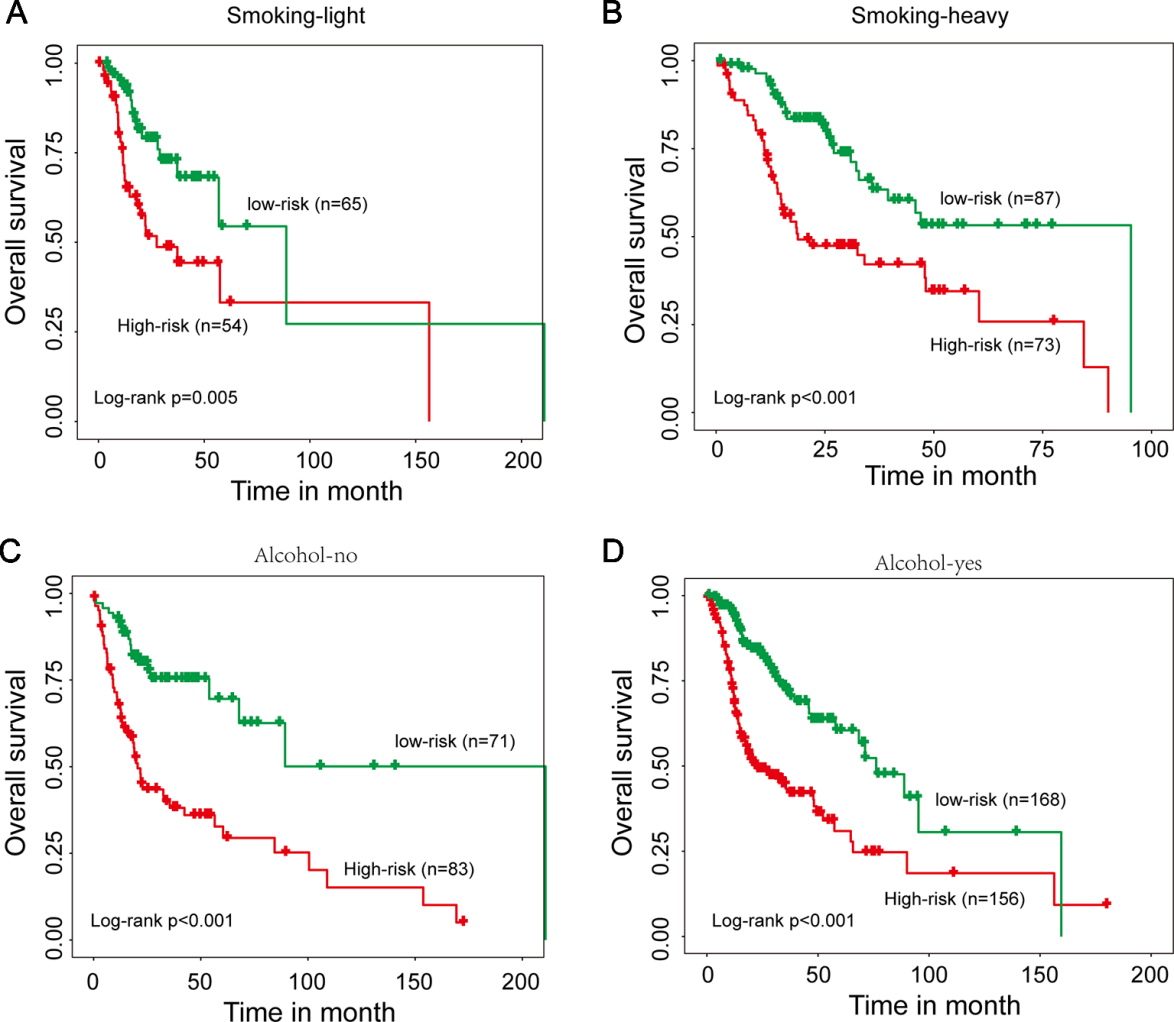
Stratification analysis for smoking and alcohol. **(A)** Kaplan–Meier survival curves of overall survival between the high-risk group and low-risk group for smoking-light patients. **(B)** Kaplan–Meier survival curves of overall survival between the high-risk group and low-risk group for smoking-heavy patients. **(C)** Kaplan–Meier survival curves of overall survival between the high-risk group and low-risk group for alcohol-no patients. **(D)** Kaplan–Meier survival curves of overall survival between the high-risk group and low-risk group for alcohol-yes patients.

We next performed a stratification analysis of smoking and alcohol. A total of 279 patients with smoking information were firstly divided into two patient data sets: smoking-light data set (n = 119) and smoking-heavy data set (n = 160). Using the 15SigRS, patients in the smoking-light data set could be subdivided into a high-risk group and low-risk group with the significantly different outcome (**Figure 5A**, p = 0.005, log-rank test). Similar results were observed when the 15SigRS was tested in the smoking-heavy data set (**Figure 5B**, p < 0.001, log-rank test). Then 478 patients with alcohol information were divided into two patient data sets: alcohol-no data set (n = 154) and alcohol-yes data set (n = 324). Using the 15SigRS, patients in the alcohol-no data set could be subdivided into the high-risk group (n = 83) and low-risk group (n = 71) with the significantly different outcome (**Figure 5C**, p < 0.001, log-rank test). Similar results were observed when the15SigRS was tested in the alcohol-yes data set (**Figure 5D**, p < 0.001, log-rank test). Multivariate and stratification analysis shows that the predictive power of the 15SigRS was independent of other clinicopathological factors for survival prediction in a patient with HNSCC.

### Performance Comparison of the 15SigRs With the Single RNA Type-Based Signatures

**Figure 6:**
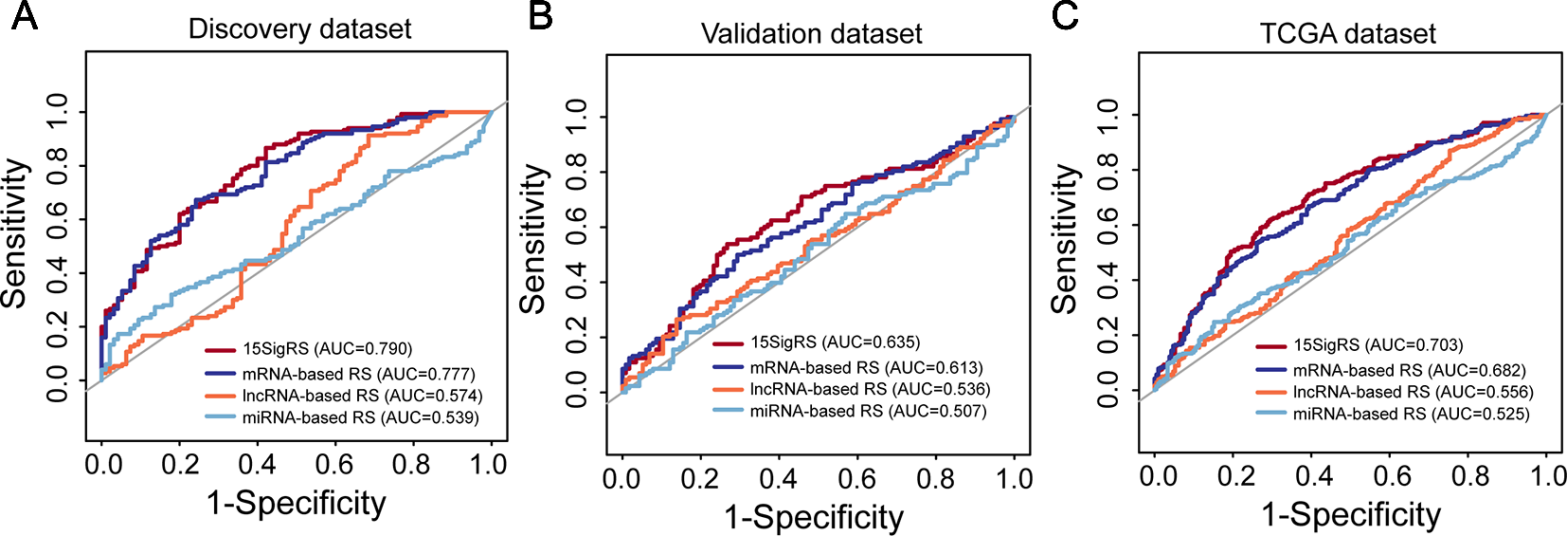
ROC analysis of the 15SigRS with the single RNA type-based signatures in the discovery data set **(A)**, validation data set **(B)**, and TCGA data set **(C)**.

We then performed a comparative analysis for predictive performance of the 15SigRS with other single RNA type-based signatures. We performed ROC analysis and computed AUCs for 15SigRS and the other three types of RNA signatures in three data sets, respectively. As shown in **Figure 6A**, the15SigRS achieved a better prediction performance with an AUC value of 0.79 in the discovery data set, which is higher than other three types RNA signatures (mRNA-based signature AUC = 0.777, lncRNA-based signature AUC = 0.574, and miRNA signature AUC = 0.539). The 15SigRS also performed well in the validation data set and TCGA data set compared with other three types of RNA signatures (**Figures 6B**, **C**). Taken together, the 15SigRS generated by our approach has a prior performance in prognostic prediction than other single RNA type-based signatures.

### Functional Characteristics of the 15SigRs

**Figure 7:**
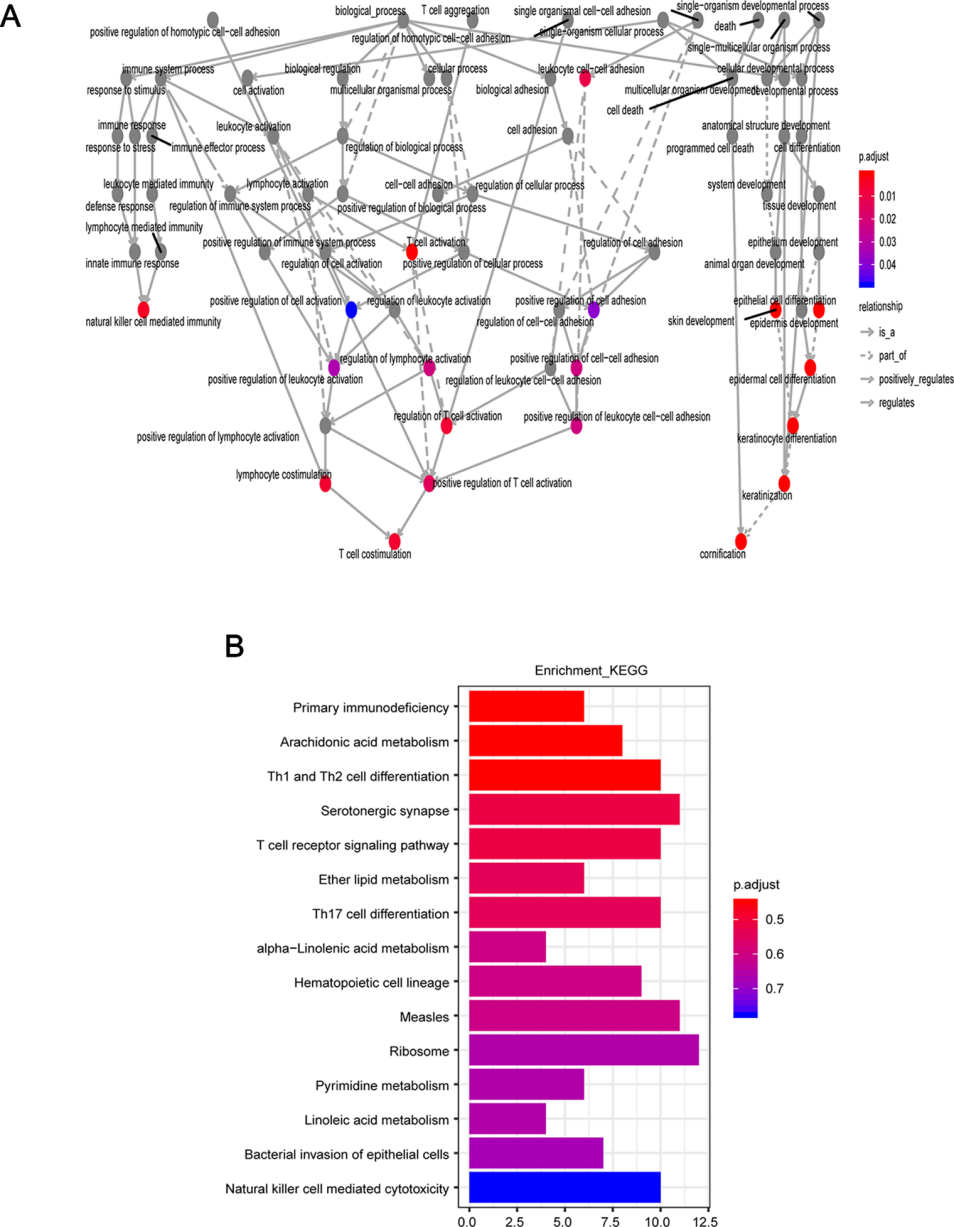
Function enrichment analysis. **(A)** GO enrichment analysis. **(B)** KEGG enrichment analysis.

To further explore the potential function of the 15SigRS, we first calculated the Pearson correlation coefficient between expression levels of mRNAs and lncRNAs in the 15SigRS and identified ranking top 5% mRNAs as lncRNA-related mRNAs. Then we performed GO and KEGG functional enrichment analysis for these lncRNA-related mRNAs. Results of GO enrichment analysis suggested that these lncRNA-related mRNAs are enriched in immune- or cell differentiation-related GO terms (**Figure 7A**). Results of KEGG enrichment analysis suggested that these lncRNA-related mRNAs are enriched in immune- or metabolism-related KEGG pathways (**Figure 7B**).

## Discussion

The molecular landscape has highlighted that HNSCC is a heterogeneous disease characterized by different molecular subgroups and clinical features (Leemans et al., 2018). Despite improvements in diagnosis and treatment for HNSCC patients, different patient subgroups with different molecular features and same TNM stage might benefit from effective personalized treatment options. Therefore, it is critical to identify reliable molecular biomarkers for distinguishing different risk patient subgroup. Although increasing efforts have been made to meet this need, previously reported gene signatures involved in only one type RNA such as mRNAs, lncRNAs, and miRNAs. Cooperative roles among different RNA molecules have been unveiled in cancer development and progression (Zhou et al., 2016a; Zhou et al., 2016b; Pan et al., 2019; Zhu et al., 2019). Therefore, in this study, we performed a systematic analysis to examine the joint predictive power of a multi-type RNA signature in the prognosis of HNSCC patients through integration analysis of mRNA, miRNA, and lncRNA expression profiles and clinical data in a large number of HNSCC patients.

Because of the limitation in available HNSCC patient data with paired mRNA profiles, miRNAs, lncRNA profiles, and clinical data, TCGA HNSCC patient data were first split randomly into two independent patient data sets for the purpose of discovery and independent validation. Then we identified 15 RNA genes (including 10 mRNAs, 4 lncRNAs, and 1 miRNA) as independent biomarkers and constructed a 15-RNA signature (15SigRS) which can classify patients into the high-risk group and low-risk group with a significantly different outcome in the discovery data set. Furthermore, the 15SigRS was further validated in the independent patient data set which revealed the performance robustness in survival prediction. Further multivariate Cox regression analysis and stratification analysis demonstrated the independence of predictive performance of the 15SigRS relative to conventional clinicopathological factors, such as age, gender, stage, grade, race, smoking, and drinking, both in discovery data set and validation data set.

Among 15 RNAs in the signature, several RNAs have been reported to be associated with cancer development and prognosis. For example, ADGRE1 encodes F4/80 antigen which was expressed in immune cells and used as a monocyte-macrophage marker in mice (Waddell et al., 2018). KRT84 has been reported to be up-regulated in squamous cell carcinoma and involved in metabolic pathways (Koringa et al., 2016). Sancisi found that CDH6 was highly expressed in thyroid tumor patients and could be as a regulator of invasiveness in thyroid tumors (Sancisi et al., 2013). The pan-cancer analysis suggested that hsa-mir-4664 was over-expressed in eight cancers (Hu et al., 2018). Low LINC00968 expression has recently reported associated with poor prognosis in breast cancers by attenuating drug resistance (Xiu et al., 2019). To gain a global view for the biological function of the 15SigRS, we performed a GO and KEGG function enrichment analysis which indicated that the 15SigRS may be involved in immune- or metabolism-related biological function.

These are several limitations in our study that need to be noted. First, only some of 15 RNAs in the 15SigRS have been experimentally studied, and other remaining RNAs should be investigated in further experiments which may provide new therapeutic target in HNSCC. Second, the 15SigRS was validated in only one independent patient data set because of data limitations, and more patient data sets were expected to validate the performance of the 15SigRS for accelerating the clinical application. Taken together, our study identified a novel multi-type RNA signature associated with the clinical outcome of HNSCC patients. This signature may be a novel independent molecular prognostic marker for selecting high-risk patients which may benefit from more individualized treatment.

## Data Availability Statement

The data analyzed in this study was obtained from the Cancer Genome Atlas (TCGA) database (https://cancergenome.nih.gov/) and Gene Expression Omnibus (GEO) database (https://www.ncbi.nlm.nih.gov/geo/query/acc.cgi?acc=GSE65858).

## Author Contributions

SW conceived and designed the experiments. SW, XD, and DX performed the experiments and analyzed the data. SW wrote the paper. All authors read and approved the final manuscript.

## Conflict of Interest

The authors declare that the research was conducted in the absence of any commercial or financial relationships that could be construed as a potential conflict of interest.
